# Combined model of radiomics, clinical, and imaging features for differentiating focal pneumonia-like lung cancer from pulmonary inflammatory lesions: an exploratory study

**DOI:** 10.1186/s12880-022-00822-5

**Published:** 2022-05-24

**Authors:** Jun-wei Gong, Zhu Zhang, Tian-you Luo, Xing-tao Huang, Chao-nan Zhu, Jun-wei Lv, Qi Li

**Affiliations:** 1 Department of Radiology, The First Affiliated Hospital of Chongqing Medical University, Chongqing, 400016 China; 2 Department of Nuclear Medicine, The First Affiliated Hospital of Chongqing Medical University, Chongqing, 400016 China; 3 Department of Radiology, The Fifth People’s Hospital of Chongqing, Chongqing, 400062 China; 4 Hangzhou YITU Healthcare Technology, Hangzhou, 310024 China; 5 Department of Radiology, The First Affiliated Hospital of Chongqing Medical University, No. 1 Youyi Road, Yu zhong District, Chongqing, 400016 China

**Keywords:** Pneumonia-like lung cancer, Pulmonary inflammation, Computed tomography, Radiomics

## Abstract

**Background:**

Only few studies have focused on differentiating focal pneumonia-like lung cancer (F-PLC) from focal pulmonary inflammatory lesion (F-PIL). This exploratory study aimed to evaluate the clinical value of a combined model incorporating computed tomography (CT)-based radiomics signatures, clinical factors, and CT morphological features for distinguishing F-PLC and F-PIL.

**Methods:**

In total, 396 patients pathologically diagnosed with F-PLC and F-PIL from two medical institutions between January 2015 and May 2021 were retrospectively analyzed. Patients from center 1 were included in the training (n = 242) and internal validation (n = 104) cohorts. Moreover, patients from center 2 were classified under the external validation cohort (n = 50). The clinical and CT morphological characteristics of both groups were compared first. And then, a clinical model incorporating clinical and CT morphological features, a radiomics model reflecting the radiomics signature of lung lesions, and a combined model were developed and validated, respectively.

**Results:**

Age, gender, smoking history, respiratory symptoms, air bronchogram, necrosis, and pleural attachment differed significantly between the F-PLC and F-PIL groups (all *P* < 0.05). For the clinical model, age, necrosis, and pleural attachment were the most effective factors to differentiate F-PIL from F-PLC, with the area under the curves (AUCs) of 0.838, 0.819, and 0.717 in the training and internal and external validation cohorts, respectively. For the radiomics model, five radiomics features were found to be significantly related to the identification of F-PLC and F-PIL (all *P* < 0.001), with the AUCs of 0.804, 0.877, and 0.734 in the training and internal and external validation cohorts, respectively. For the combined model, five radiomics features, age, necrosis, and pleural attachment were independent predictors for distinguishing between F-PLC and F-PIL, with the AUCs of 0.915, 0.899, and 0.805 in the training and internal and external validation cohorts, respectively. The combined model exhibited a better performance than had the clinical and radiomics models.

**Conclusions:**

The combined model, which incorporates CT-based radiomics signatures, clinical factors, and CT morphological characteristics, is effective in differentiating F-PLC from F-PIL.

## Background

In recent years, the diagnosis and treatment of lung cancer have improved significantly [1, 2]. However, it remains the leading cause of cancer-related deaths globally [3]. Computed tomography (CT) scan is an indispensable tool for the diagnosis of lung cancer. The condition has different appearances on CT scan. That is, it can present as a solitary nodule/mass, localized or diffuse parenchymal consolidation, or multifocal lesions. In clinical practice, if lung cancer manifests as focal consolidation, it is easily misdiagnosed as pulmonary inflammatory lesions due to the poor comprehension of its imaging findings [4, 5]. The results of histopathological examination such as bronchoscopy and CT-guided percutaneous lung biopsy can help physicians obtain an accurate diagnosis and select the best treatment strategy [6]. However, these methods are invasive, and they may occasionally obtain an incorrect pathological diagnosis due to insufficient histological samples [7, 8]. Therefore, a noninvasive and reproducible quantitative method is highly preferred in determining whether the lesions are benign or malignant.

Radiomics is a computerized quantitative image analysis method that uses computational algorithms to extract a large number of features from radiological medical images [9, 10]. It can quantify the heterogeneity of tumors and has advantages including reproducibility and noninvasiveness without time and space limitations [11, 12]. Unlike some histopathological methods that can only provide information about a part of the lesion, radiomics can obtain more information about the whole lesion [13, 14]. In recent years, radiomics has played an increasingly important role in differentiating benign and malignant lesions and in evaluating the curative effect and prognosis of tumors [15, 16]. However, to the best of our knowledge, only few studies have focused on the differential diagnosis of focal pneumonia-like lung cancer (F-PLC) and focal pulmonary inflammatory lesion (F-PIL) using radiomics.

Therefore, the current exploratory study aimed to develop a model incorporating CT-based radiomics signatures, clinical factors, and CT morphological characteristics to differentiate F-PLC from F-PIL. Moreover, its efficacy was validated.

## Material and methods

### Patients

**Fig. 1:**
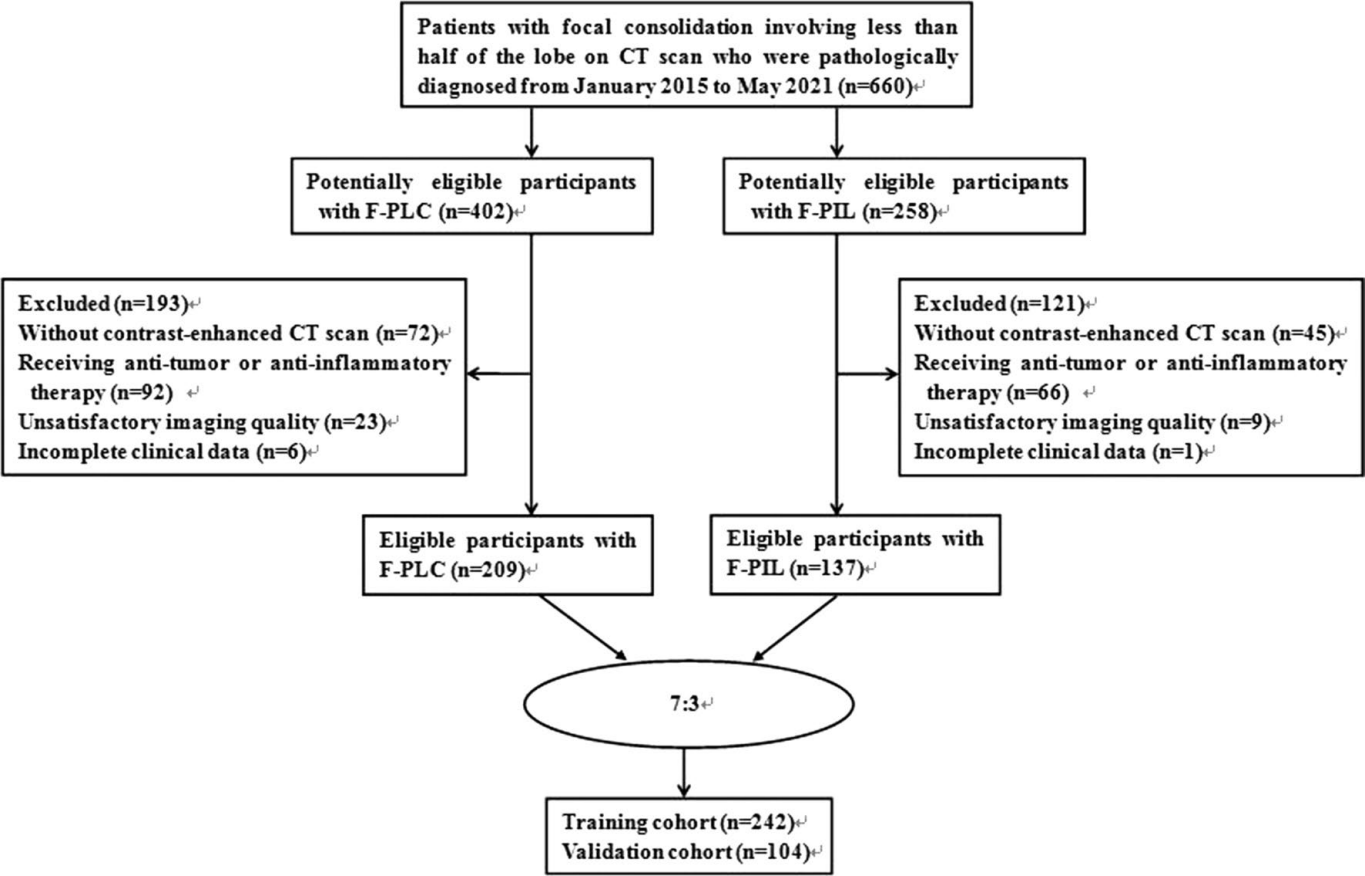
Flow chart of patient selection

The ethics committee of the First Affiliated Hospital of Chongqing Medical University approved this retrospective study. All methods were carried out in accordance with relevant guidelines and regulations. Informed consent (written consent) was obtained from all individual participants included in the study. In this research, F-PLC was defined as a special type of lung cancer presenting as focal consolidation involving less than half of the lobe. Meanwhile, F-PIL refers to focal inflammatory consolidation affecting less than half of the lobe. The inclusion criteria were as follows: (1) patients with a pathological diagnosis confirmed via surgical resection; (2) patients who underwent chest CT scan at our institutes; (3) CT results indicated a solitary and focal consolidation, characterized by an increased density of lung parenchyma with obscuration of the underlying vessels, with a polygonal shape (e.g., triangular, rectangular, or trapezoidal) and the largest slice involving less than half the area of a lobe on axial CT images; (4) patients with interval of < 1 month between CT imaging and subsequent pathological analysis. Meanwhile, the exclusion criteria were as follows: (1) patients who did not undergo chest contrast-enhanced CT scan; (2) patients with unsatisfactory imaging quality due to respiratory motion artifact; (3) patients who received any anti-tumor or anti-inflammatory therapy before initial chest CT scanning; (4) patients with incomplete clinical data. In total, 346 (193 men and 153 women; mean age: 61.5 ± 14 [range: 22–86] years) patients diagnosed between January 2015 and May 2021 at center 1 were included. Among them, 209 presented with F-PLC and 137 with F-PIL. We randomized the patients into two groups, with a ratio of 7:3 (242 patients in the training cohort and 104 patients in the internal validation cohort) (Fig. 1). Moreover, we included 50 consecutive patients (29 men and 21 women; mean age: 64 ± 13.5 [range: 38–87] years) diagnosed at center 2 in the external validation cohort. Among them, 27 presented with F-PLC and 23 with F-PIL.

### CT image acquisition and morphological features analysis

All chest contrast-enhanced CT examinations were performed using Discovery CT750HD (GE Healthcare) and Optima CT660 (GE Healthcare). All patients underwent CT scan in a supine position at the end of inspiration during a single breath hold to prevent respiratory motion artifacts. The CT scanning parameters were as follows: tube voltage, 100–130 kVp; tube current, 100–250 mA; slice thickness, 5.0 mm; and slice interval, 5.0 mm. Nonionic iodinated contrast medium (iohexol 300 mg iodine/mL; Omnipaque, GE Healthcare) at a dose of 1.5 mL/kg was administered at a flow rate of 3.0 mL/s using a dual-high-pressure injector via the antecubital vein. Then, 50 mL of saline solution was injected. The arterial and delayed phases were triggered at 30 and 120 s, respectively.

Two thoracic radiologists (with 13 and 6 years of experience in chest imaging, respectively) reviewed all CT images in a PACS workstation (Vue PACS, Carestream) together. In cases of disagreement, a consensus was reached via discussion. The lung window (window width, 1600 HU; window level, − 600 HU) and mediastinal window (window width, 450 HU; window level, 50 HU) of CT images were used for assessment. CT morphological features including lesion size (maximum diameter of the lesion on axial CT images), margin (well-defined, with a clear border definition; ill-defined, with a partial or completely blurred border), spiculation (linear strands extending from the nodule or mass margin into the lung parenchyma without reaching the pleural surface), air bronchogram (tubelike or branched air structure within the lesion), pleural attachment (lesion attaching to the pleura including the fissure and lesion margin obscured by the pleura), necrosis (unenhanced areas with clear boundaries within the lesion), calcification, lymphadenopathy (hilar or mediastinal lymph nodes with short-axis diameter larger than 1 cm), and pleural effusion were evaluated.

### Comparison of clinical and CT features between both groups

The clinical and CT morphological features including age, gender, smoking history, respiratory symptoms, lesion size, margin, spiculation, air bronchogram, necrosis, calcification, lymphadenopathy, pleural attachment, and pleural effusion between the F-PLC and F-PIL groups in center 1 were compared.

### Model establishment and performance evaluation

#### Establishment of clinical model

The clinical model was constructed by incorporating clinical and CT morphological features that differed significantly between the F-PLC and F-PIL groups in center 1 using the multivariate logistic regression analysis.

#### Establishment of radiomics model

**Fig. 2:**
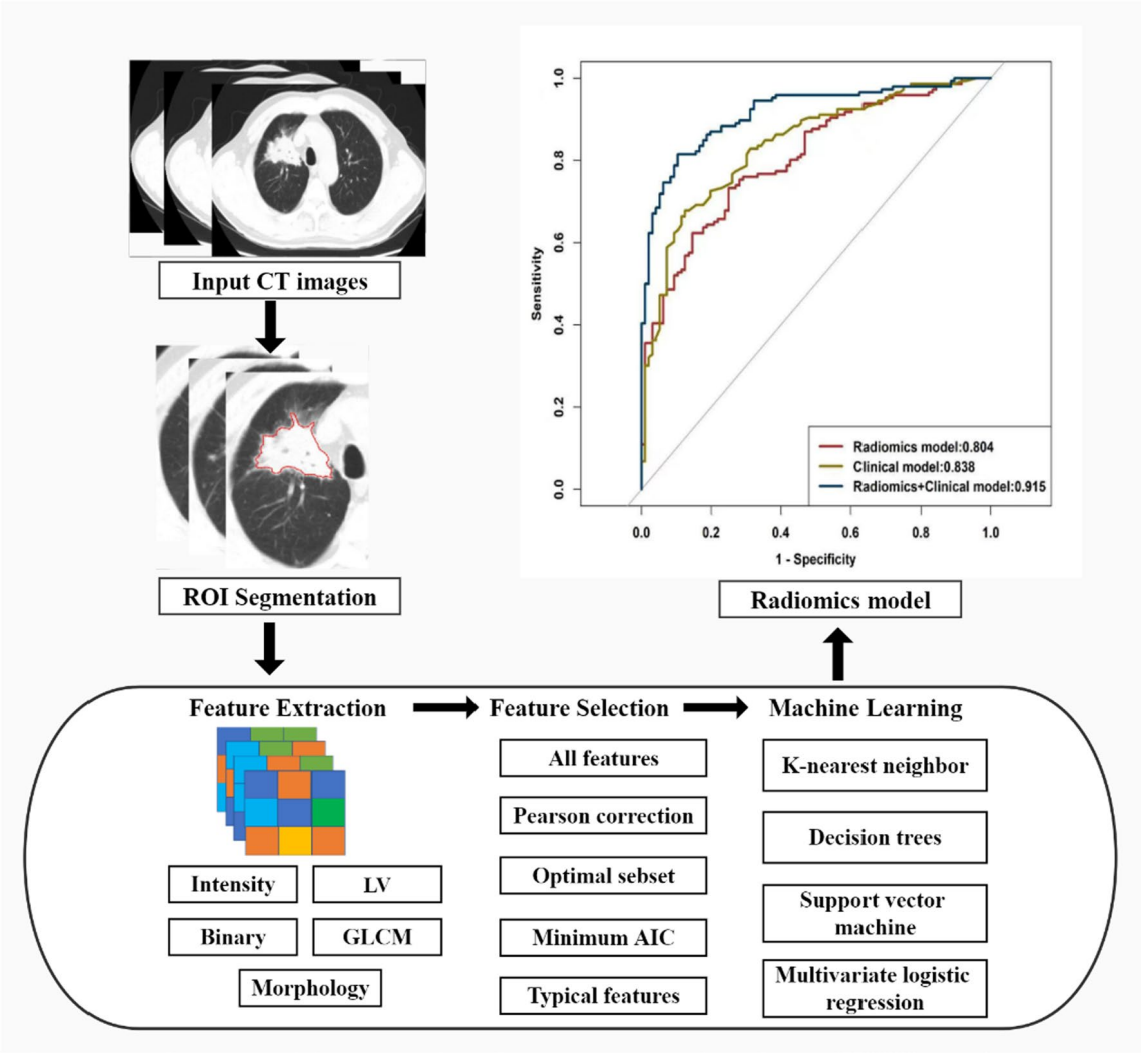
Flow chart of radiomics implementation in this study

The region of interest (ROI) on non-contrast-enhanced CT images with lung window was manually segmented by one radiologist (with 6 years of work experience in thoracic imaging) who did not know the clinical and pathological information of patients using the ITK software (version 2.2.0, http://www.itksnap.org/pmwiki/pmwiki.php). Three ROIs were cautiously drawn along the margin of the lesion in the largest layer and the adjacent upper and lower layers from axial CT images, covering the whole contour of the solid component of the lesion. Figure 2 shows the radiomics analysis flowchart. To ensure consistency, these delineations were conducted three times. To evaluate intra observer repeatability, the observer repeated the ROI delineation 4 weeks after the first assessment. Thereafter, the intra-class correlation coefficients (ICCs) were calculated to evaluate the stability and reproducibility of feature extraction. These features with ICC values > 0.75 were included in this study.

Radiomics feature extraction was performed using Pyradiomic implemented in Python (https://pyradiomic.readthedocs.io/en/latest/), which can extract radiomics features from CT images with a large panel of engineered hard-coded feature algorithm. As shown in Fig. 2, the steps for selecting radiomics features and signature building were as follows: first, we imported the CT images into the software with Digital Imaging and Communications in Medicine files (planning CT images with GTV ring). The images were preprocessed and segmented, and the resolution feature matrix was normalized. Second, we compared the similarity of each feature pair. If the Pearson correlation coefficient of a feature pair was > 0.90, one of them will be deleted. Third, the optimal subset method with the Akaike information criterion (AIC) information criteria were combined to select features. Finally, we established the logistic regression radiomics model using a combination of features under the minimum AIC correspondence.

#### Establishment of combined model

The combined model, which integrated CT-based radiomics signatures, clinical factors, and CT morphological features, for differentiating F-PLC from F-PIL was developed via a multivariate logistic regression analysis.

#### Performance evaluation of the three models

The receiver operating characteristic curve (ROC) and the area under the curve (AUC), accuracy, sensitivity, and specificity were used to evaluate the performance of three models in the training and internal and external validation cohorts, respectively.

### Statistical analysis

Statistical analysis was performed using the Statistical Package for the Social Sciences software for Windows (version 19.0, IBM, Armonk, NY, USA) and R software (version 3.6.1; http://www.Rproject.org). Quantitative data with a normal distribution were expressed as mean ± standard deviation, whereas those with a non-normal distribution were presented as medians ± interquartile ranges. Meanwhile, categorical variables were expressed as numbers and percentages. The Chi-square test, Two-independent-samples Student’s t-test, and Mann–Whitney U test were used in the univariate analysis. DeLong test was performed to compare the AUCs of three models in the internal and external validation cohorts. A two-sided *P*-value of < 0.05 was considered statistically significant.

## Results

### Clinical and CT morphological features between the F-PLC and F-PIL groups

**Fig. 3:**
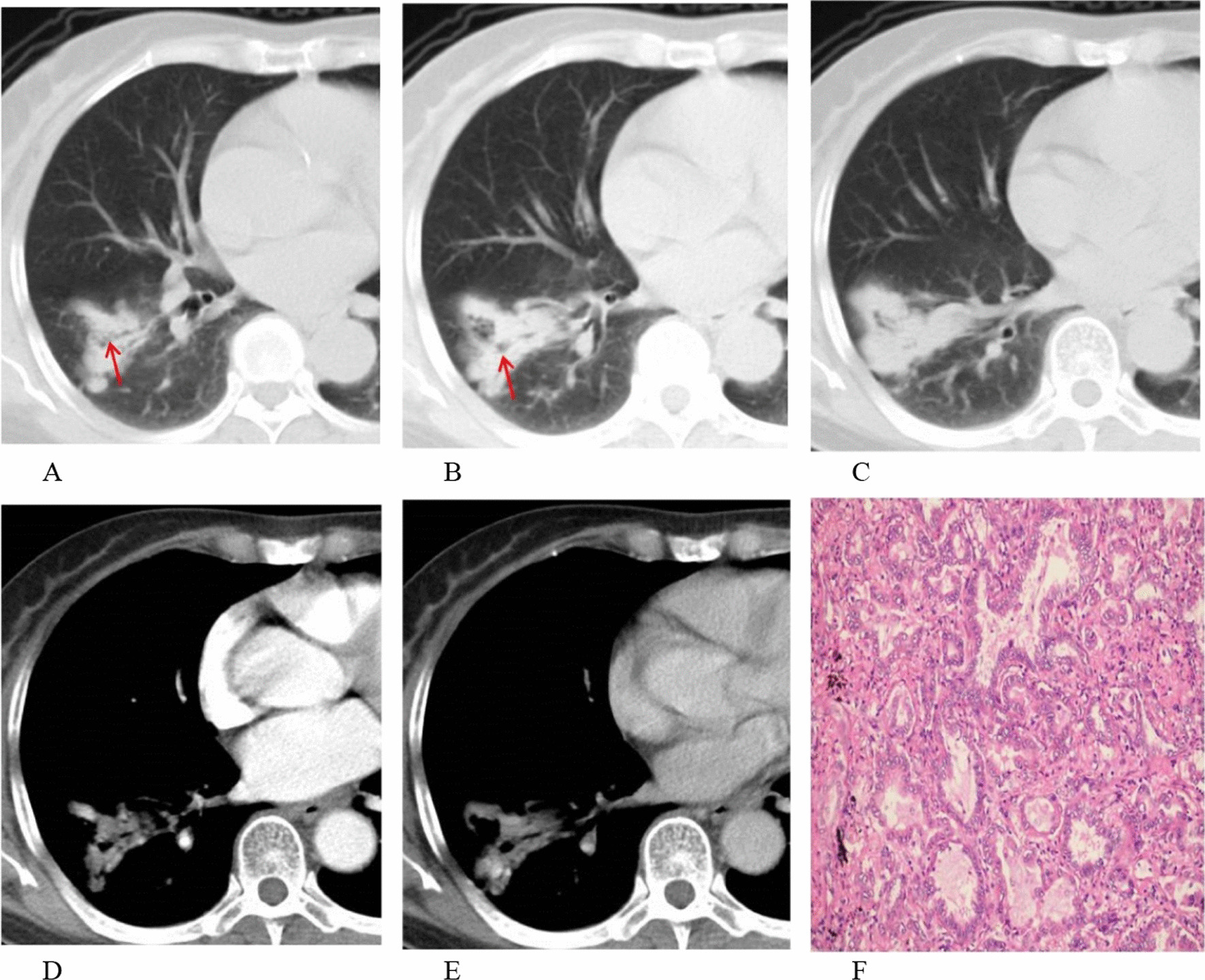
A 68-year-old woman with invasive adenocarcinoma. **A**–**C** Air bronchogram was observed in the lung window (red arrow). **D**, **E** The uneven enhancement of the lesion in the largest layer was found in the mediastinal window. **F** Photomicrograph (hematoxylin and eosin staining, × 200) confirming invasive adenocarcinoma with an acinar-predominant pattern

**Fig. 4:**
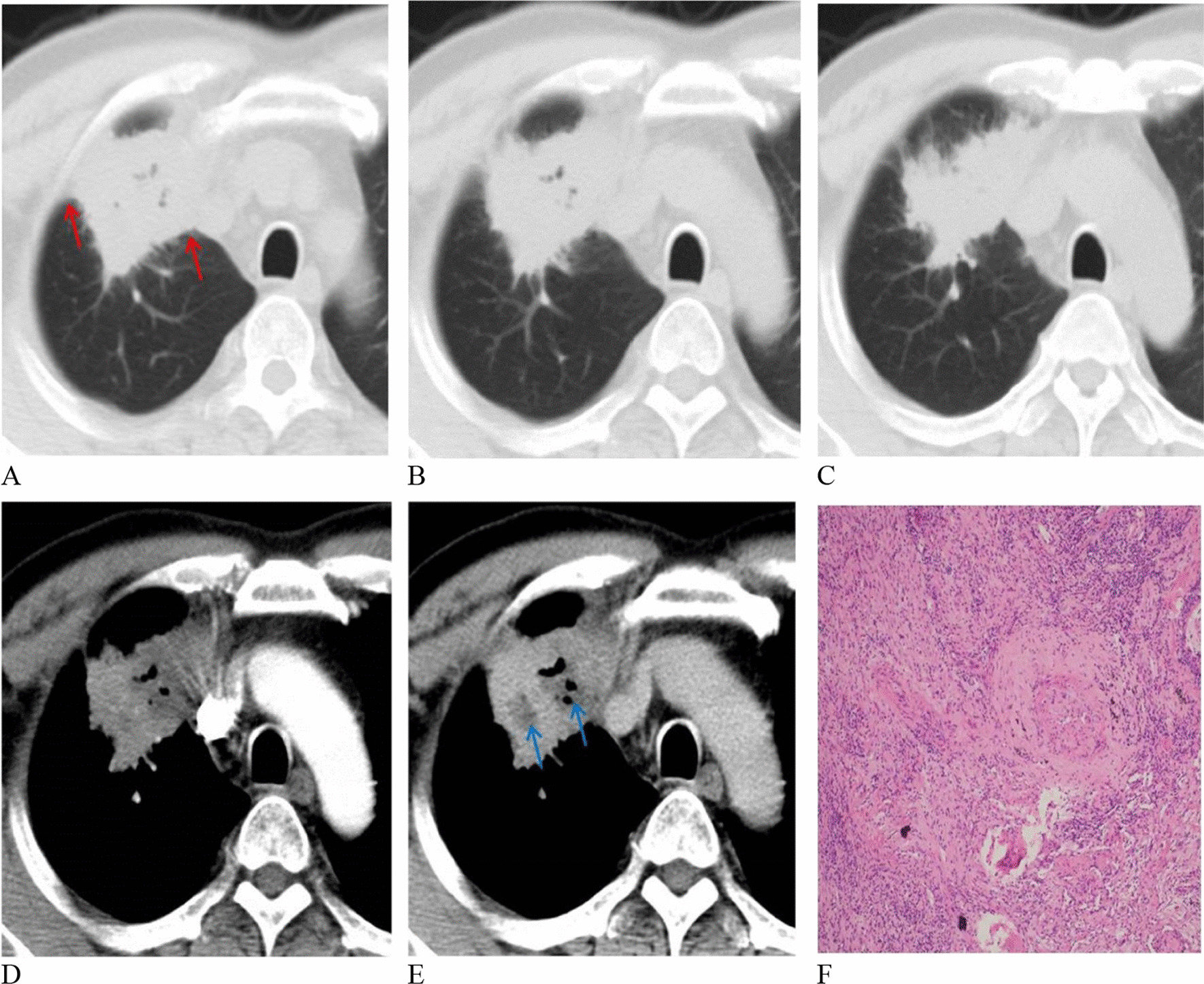
A 53-year-old woman with inflammatory pseudotumor. **A**–**C** Pleural attachment was observed in the lung window (red arrow). **D**, **E** Lesion necrosis in the largest layer was found in the mediastinal window (blue arrow). **F** Photomicrograph (hematoxylin and eosin staining, × 100) confirming inflammatory pseudotumor

This study enrolled 346 (193 men and 153 women) patients from center 1, including 209 with F-PLC and 137 with F-PIL. The F-PLC group included 185 patients with adenocarcinoma, 21 with squamous cell carcinoma, and 3 with adeno-squamous carcinoma. The F-PIL group comprised 45 patients with nonspecific pulmonary inflammation, 44 with inflammatory pseudotumor, 32 with tuberculosis, 9 with organizing pneumonia, 6 with aspergillus infection, and 1 with lung abscess. For surgical resection methods, 209 patients with F-PLC and 84 patients with F-PIL underwent lobectomy, while 53 patients with F-PIL underwent sub-lobectomy. The patients with F-PLC were older than those with F-PIL (*P* < 0.001). Compared to patients with F-PIL, those with F-PLC were more frequently females, non-smokers and presented without respiratory symptoms (all *P* < 0.05). In total, the two groups were compared on the basis of 9 CT morphological features. Among them, air bronchogram was more frequently observed in the F-PLC than in the F-PIL group (*P* < 0.05, Fig. 3). The F-PIL group more commonly presented with necrosis and pleural attachment than did the F-PLC group (all *P* < 0.001, Fig. 4). However, the two groups did not significantly differ in terms of lesion size, margin, spiculation, calcification, lymphadenopathy, and pleural effusion (all *P* > 0.05) (Table 1).

**Table 1:** Clinical and CT morphological features between the F-PLC and F-PIL groups

Characteristics	F-PLC group (n = 209)	F-PIL group (n = 137)	*p*-Value
Age (years)			< 0.001^a^
Median ± interquartile range	64 ± 12	55 ± 16	
Gender			< 0.001^b^
Male	100 (47.85%)	93 (67.88%)	
Female	109 (52.15%)	44 (32.12%)	
Smoking history			0.024^b^
Smokers	84 (40.19%)	72 (52.55%)	
Non-smokers	125 (59.81%)	65 (47.45%)	
Respiratory symptoms^d^			< 0.001^b^
With symptoms	126 (60.29%)	113 (82.48%)	
Without symptoms	83 (39.71%)	24 (17.52%)	
Lesion size (mm)			0.067^c^
Mean ± standard deviation	43.17 ± 18.47	46.75 ± 16.57	
Margin			0.302^b^
Well-defined	138 (66.03%)	83 (60.58%)	
Ill-defined	71 (33.97%)	54 (39.42%)	
Spiculation	14 (6.70%)	9 (6.57%)	0.962^b^
Air bronchogram	59 (28.23%)	19 (13.87%)	0.002^b^
Necrosis	17 (8.13%)	61 (44.53%)	< 0.001^b^
Calcification	13 (6.22%)	15 (10.95%)	0.115^b^
Lymphadenopathy	66 (31.58%)	35 (25.55%)	0.227^b^
Pleural attachment	103 (49.28%)	118 (86.13%)	< 0.001^b^
Pleural effusion	13 (6.22%)	6 (4.38%)	0.462^b^

*CT* computed tomography, *F-PLC* focal pneumonia-like lung cancer, *F-PIL* focal pulmonary inflammatory lesion

^a^Mann–Whitney U test

^b^Chi-square test

^c^Two-independent-samples Student’s t-test

^d^Respiratory symptoms including fever, cough, sputum, blood in sputum, hemoptysis, and chest pain

### Performance of clinical model

**Fig. 5:**
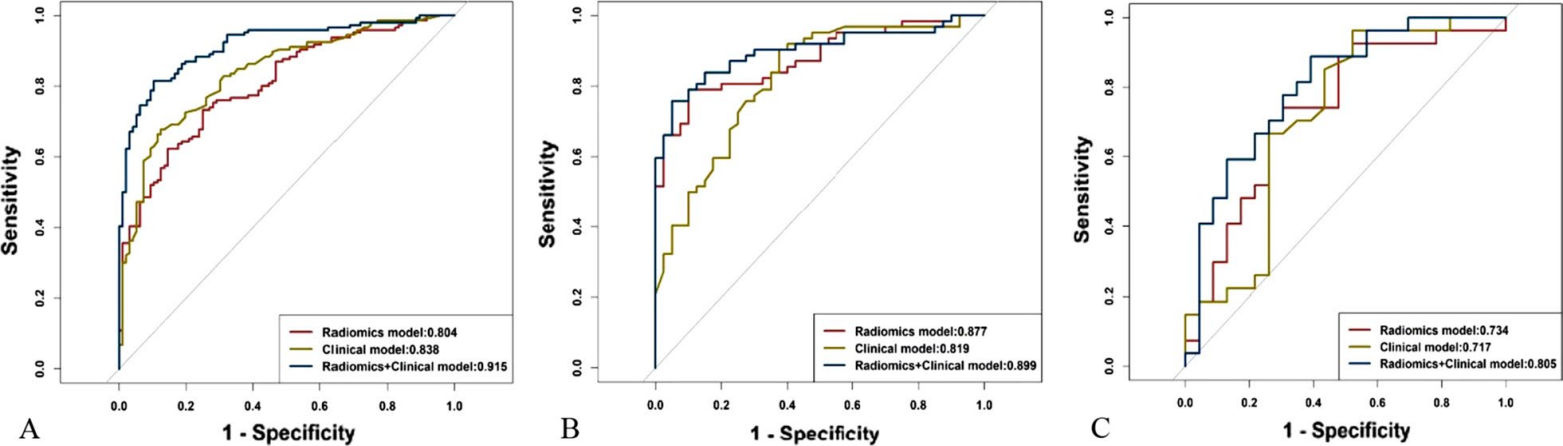
The receiver operating characteristic curve analyses in the training cohort (**A**), internal validation cohort (**B**), and external validation cohort (**C**)

The clinical model showed that age, necrosis, and pleural attachment were the most effective factors for differentiating F-PIL from F-PLC with the AUCs of 0.838, 0.819, and 0.717 in the training and internal and external validation cohorts, respectively. The accuracy, sensitivity, and specificity were 0.785, 0.801, and 0.760 in the training cohort, 0.731, 0.603, and 0.902 in the internal validation cohort, and 0.720, 0.852, and 0.609 in the external validation cohort, respectively (Table 2, Fig. 5).

**Table 2:** Performance of the clinical, radiomics, and combined models

Group	AUC (95% CI)	Cut-off	Accuracy	Sensitivity	Specificity
Training cohort (n = 242)					
Clinical model	0.838 (0.788–0.889)	0.626	0.785	0.801	0.760
Radiomics model	0.804 (0.75–0.858)	0.526	0.740	0.733	0.750
Combined model	0.915 (0.88–0.95)	0.639	0.847	0.815	0.896
Internal validation cohort (n = 104)					
Clinical model	0.819 (0.738–0.901)	0.740	0.731	0.603	0.902
Radiomics model	0.877 (0.812–0.942)	0.525	0.833	0.790	0.900
Combined model	0.899 (0.839–0.96)	0.653	0.833	0.758	0.950
External validation cohort (n = 50)					
Clinical model	0.717 (0.562–0.871)	0.439	0.720	0.852	0.609
Radiomics model	0.734 (0.59–0.879)	0.364	0.720	0.704	0.739
Combined model	0.805 (0.681–0.929)	0.283	0.760	0.889	0.609

*AUC* area under the ROC curve, *CI* confidence interval

### Performance of radiomics model

In total, 1583 radiomics features were selected from patients with F-PLC and F-PIL. After ranking these features, the five best-performing significant radiomics features were square first-order robust mean absolute deviation, logarithm gldm large dependence high gray level emphasis, wavelet HLL first-order median, square root glszm low gray level zone emphasis, and logarithm glcm lmc1. They were found to be significantly associated with the identification of both F-PLC and F-PIL (all *P* < 0.001). The AUC, accuracy, sensitivity, and specificity were 0.804, 0.740, 0.733, and 0.750 in the training cohort, 0.877, 0.833, 0.790, and 0.900 in the internal validation cohort, and 0.734, 0.720, 0.704, and 0.739 in the external validation cohort, respectively (Table 2, Fig. 5).

### Performance of combined model

The combined model indicated that the five best-performing significant radiomics features, age, necrosis, and pleural attachment were independent predictors for differentiating F-PLC form F-PIL. The AUC, accuracy, sensitivity, and specificity were 0.915, 0.847, 0.815, and 0.896 in the training cohort, 0.899, 0.833, 0.758, and 0.950 in the internal validation cohort, and 0.805, 0.760, 0.889, and 0.609 in the external validation cohort, respectively (Table 2, Fig. 5).

### Comparation of the AUCs of three models in the validation cohorts

For the internal validation cohort, the AUC of combined model was significantly larger than that of clinical model (*P* < 0.05); however, no significant difference in the AUC was observed between combined and radiomics models (*P* > 0.05). For the external validation cohort, no significant differences in the AUC were observed among the three models (*P* > 0.05).

## Discussion

Similar to F-PIL, F-PLC manifests as a localized consolidation on CT images. Therefore, differentiating F-PLC from F-PIL via CT scan is often challenging particularly if patients with F-PIL do not present with typical clinical symptoms or if the lesions are not absorbed after anti-inflammatory therapy. In the current study, 68 (32.5%) of 209 patients with F-PLC were misdiagnosed with inflammatory lesions on initial CT scan, while 56 (40.9%) of 137 patients with F-PIL were misdiagnosed with cancers. Radiomics focuses on the extraction of sub-visual, yet quantitative, imaging features from radiological images [10, 17]. In recent years, numerous studies have shown that radiomics can distinguish the pathological types of lung cancer [18–20], reflect the association between intra-tumoral heterogeneity and underlying gene expression patterns [21], and distinguish adenocarcinomas from granulomas [22, 23]. Therefore, this research developed and validated a combined model incorporating CT-based radiomics signatures, clinical factors, and CT morphological features for differentiating F-PLC from F-PIL.

This study first compared the clinical and CT morphological features of the F-PLC and F-PIL groups. Results showed that age, gender, smoking history, clinical symptoms, and three CT morphological features including air bronchogram, necrosis, and pleural attachment differed significantly between the two groups. Then, we further established a clinical model based on aforementioned differential features with the AUCs of 0.838, 0.819, and 0.717 in the training and internal and external validation cohorts, respectively. This model suggested that age, necrosis, and pleural attachment were the most effective factors for differentiating F-PIL from F-PLC. Next, we extracted radiomics features from CT images. The five best-performing significant radiomics signatures were used to establish the radiomics model. The AUCs in the training and internal and external validation cohorts were 0.804, 0.877, and 0.734, respectively, thereby indicating radiomics can distinguish F-PLC from F-PIL. Previous studies [24, 25] have shown that radiomics signatures play an important role in differentiating lung cancer from inflammatory lesions, and our study had similar results. Finally, we incorporated CT-based radiomics signatures, clinical factors, and CT morphological features to establish a combined model. In this model, five best-performing radiomics features, age, necrosis, and pleural attachment were found to be independent predictors for distinguishing between F-PLC and F-PIL. A previous study [26] has shown that patients with lung cancers were older than those with inflammatory lesions, which is similar to our findings. Regarding radiological features, we found that necrosis and pleural attachment were highly indicative of F-PIL, and this finding can be supported by the results of prior studies [5, 26, 27]. Generally, necrotizing infectious lung lesions are closely correlated with inflammatory response caused by microbial infection. Meanwhile, necrotizing cancer is associated with continuous angiogenesis and chronic ischemic injury, which likely occur in large tumors and, accordingly, are seldom observed in F-PLC with a localized range [28, 29]. Inflammatory exudation can easily spread to the subpleural area via the alveolus if the lesion is located in the lung periphery, causing the lesion to attach to the pleura. This phenomenon may be associated with the high occurrence of pleural attachment in F-PIL. However, lung cancer seldom attaches to the pleura due to tumor mesenchymal contraction, which may result in pleural retraction [30]. This model was effective in differentiating F-PLC from F-PIL, with AUCs of 0.915, 0.889, and 0.805 in the training and internal and external validation cohorts, respectively. Zhang et al. [27] showed that the sensitivity, specificity, and AUC of thin-section CT-based radiomics in differentiating focal organizing pneumonia from peripheral adenocarcinoma were 85.3%, 89.7%, and 0.956, respectively. This finding was consistent with our results. Furthermore, we found a relative drop in performance in the external validation cohort. We think the small number of patients in the external validation cohort and lacking evaluation of the heterogeneity of patients in each cohort due to the small sample size may attribute to this result. Future studies with larger sample sizes are needed to substantiate our findings.

The current study had several limitations. First, the usefulness of the combined model is limited to the differentiation of F-PLC and F-PIL which manifested as localized consolidation involving < 50% of the area of a lobe on CT and not applicable to that of all benign and malignant lung lesions. Second, all data were collected from two institutions. Nevertheless, we will perform a multicenter study with larger sample sizes to improve the performance of the model.

## Conclusions

The combined model, which incorporates CT-based radiomics signatures, clinical factors, and CT morphological features, is effective in differentiating F-PLC from F-PIL.

## Data Availability

The datasets used and/or analyzed during the current study available from the corresponding author on reasonable request.

## Abbreviations

F-PLC: Focal pneumonia-like lung cancer
F-PIL: Focal pulmonary inflammatory lesion
CT: Computed tomography
AUC: Area under the curve
PACS: Picture archiving and communication systems
HU: Hounsfield units
ROI: Region of interest
ROC: Receiver operating characteristic
ITK: The insight segmentation and registration toolkit
LADC: Invasive lung adenocarcinoma
AIC: Akaike information criterion
CI: Confidence interval
